# Anaerobic oxidation of ammonium and short-chain gaseous alkanes coupled to nitrate reduction by a bacterial consortium

**DOI:** 10.1093/ismejo/wrae063

**Published:** 2024-04-16

**Authors:** Mengxiong Wu, Xiawei Liu, J Pamela Engelberts, Gene W Tyson, Simon J McIlroy, Jianhua Guo

**Affiliations:** Australian Centre for Water and Environmental Biotechnology (ACWEB, formerly AWMC), The University of Queensland, St Lucia, QLD 4072, Australia; Australian Centre for Water and Environmental Biotechnology (ACWEB, formerly AWMC), The University of Queensland, St Lucia, QLD 4072, Australia; Centre for Microbiome Research, School of Biomedical Sciences, Queensland University of Technology (QUT), Translational Research Institute, Woolloongabba, QLD 4102, Australia; Centre for Microbiome Research, School of Biomedical Sciences, Queensland University of Technology (QUT), Translational Research Institute, Woolloongabba, QLD 4102, Australia; Centre for Microbiome Research, School of Biomedical Sciences, Queensland University of Technology (QUT), Translational Research Institute, Woolloongabba, QLD 4102, Australia; Australian Centre for Water and Environmental Biotechnology (ACWEB, formerly AWMC), The University of Queensland, St Lucia, QLD 4072, Australia

**Keywords:** anaerobic propane and butane oxidation, anammox, dissimilatory nitrate reduction to ammonium, “Ca. A. nitratireducens”

## Abstract

The bacterial species “*Candidatus* Alkanivorans nitratireducens” was recently demonstrated to mediate nitrate-dependent anaerobic oxidation of short-chain gaseous alkanes (SCGAs). In previous bioreactor enrichment studies, the species appeared to reduce nitrate in two phases, switching from denitrification to dissimilatory nitrate reduction to ammonium (DNRA) in response to nitrite accumulation. The regulation of this switch or the nature of potential syntrophic partnerships with other microorganisms remains unclear. Here, we describe anaerobic multispecies cultures of bacteria that couple the oxidation of propane and butane to nitrate reduction and the oxidation of ammonium (anammox). Batch tests with ^15^N-isotope labelling and multi-omic analyses collectively supported a syntrophic partnership between “*Ca.* A. nitratireducens” and anammox bacteria, with the former species mediating nitrate-driven oxidation of SCGAs, supplying the latter with nitrite for the oxidation of ammonium. The elimination of nitrite accumulation by the anammox substantially increased SCGA and nitrate consumption rates, whereas it suppressed DNRA. Removing ammonium supply led to its eventual production, the accumulation of nitrite, and the upregulation of DNRA gene expression for the abundant “*Ca.* A. nitratireducens”. Increasing the supply of SCGA had a similar effect in promoting DNRA. Our results suggest that “Ca. A. nitratireducens” switches to DNRA to alleviate oxidative stress caused by nitrite accumulation, giving further insight into adaptability and ecology of this microorganism. Our findings also have important implications for the understanding of the fate of nitrogen and SCGAs in anaerobic environments.

## Introduction

Atmospheric concentrations of short-chain gaseous alkane (SCGA; including ethane, propane, and butane) have significantly increased in recent years due to anthropogenic activities, such as intensive oil and natural gas extractions [1, 2]. These SCGAs are recognized as indirect greenhouse gases and significant air pollutants, which have negative impacts on air quality and global climate [3, 4]. Microbial oxidation of SCGAs plays an important role in reducing SCGA emissions to the atmosphere, thus attracting significant attention in recent years [5, 6]. Sulfate was the first electron acceptor demonstrated to drive anaerobic oxidation of SCGAs. Sulfate-reducing bacteria (SRB) oxidize SCGAs via the fumarate addition pathway, coupled to the direct reduction of sulfate to sulfide [7]. Anaerobic SCGA oxidizing archaea activate SCGAs with divergent methyl coenzyme M reductase-like complexes, in syntrophic consortia with SRB [8, 9]. Recently, the coupling of anaerobic SCGA oxidation to nitrate reduction was also proposed to be mediated via the fumarate addition pathway by an uncultured bacterial species “*Candidatus* Alkanivorans nitratireducens” belonging to the class of Symbiobacteriia [10, 11]. These processes represent previously overlooked SCGA sinks and links between the global carbon and nitrogen cycles.

During the processes of anaerobic oxidation of SCGAs coupled to nitrate reduction, nitrite is consistently accumulated (up to 1.4 mmol N/L) [ 10, 11]. We infer this is mainly due to the lack of genes encoding nitric oxide-producing nitrite reductase (*nirS/K*) in the complete genome of “*Ca.* A. nitratireducens”. Nitrite has been found to be toxic to a range of microorganisms such as anaerobic methanotrophic bacteria “*Candidatus* Methylomirabilis oxyfera”, methanogens, and phosphate-accumulating bacteria, even at concentrations below 1 mmol N/L [12–14]. Therefore, it is likely the accumulated nitrite strongly inhibits the metabolism of “*Ca.* A. nitratireducens” (limiting rates to 0.02–0.03, 0.05–0.16, and 0.08–0.87 mmol/L/d for propane, butane, and nitrate, respectively [10, 11]). Anaerobic ammonium oxidizing (anammox) bacteria have the unique ability to consume nitrite using ammonium as an electron donor [15], thus being ideal autotrophic nitrite scavengers. Indeed, anammox bacteria have been proved to form syntrophic partnerships with the archaeon “*Candidatus* Methanoperedens nitroreducens” and bacteria affiliated with the family *Geobacteraceae*, removing nitrite generated from the anaerobic methane- and cyclohexane-driven nitrate reduction processes [16, 17]. Whether a similar partnership between “Ca. A. nitratireducens” and anammox can be sustained during nitrate driven SCGA-oxidation remains to be demonstrated.

Dissimilatory nitrate reduction to ammonium (DNRA), which converts nitrate into ammonium via nitrite, is an important component of nitrogen cycling [18]. It is suggested DNRA plays a substantial role in nitrate consumption in coastal and cropland soil ecosystems [19, 20]. During the nitrate-dependent anaerobic oxidation of SCGA processes, “*Ca.* A. nitratireducens” mediate both denitrification and DNRA, generating dinitrogen gas and ammonium [10, 11]. However, it remains unclear how “Ca. A. nitratireducens” regulate the switch between dinitrogen gas and ammonium production. Previous studies revealed that DNRA is mainly regulated by ratios of C/N ratio, NO_2_^−^/NO_3_^−^ ratio, and S^2−^ concentrations [18]. High C/N and NO_2_^−^/NO_3_^−^ ratios, and high S^2−^ concentrations generally select for DNRA [21–23]. In our previous studies, we observed DNRA primarily occurred when nitrite was accumulated in the SCGAs-fed reactors [10, 11], suggesting nitrite is likely a key factor triggering DNRA. Therefore, we hypothesize that the immediate removal of nitrite by the anammox processes will lead to suppression of the DNRA by “*Ca.* A. nitratireducens”.

Herein, the aim of this study was to understand underlying mechanisms that allow “*Ca.* A. nitratireducens” to switch between denitrification and DNRA. To this end, we established two anaerobic bioreactors coupling nitrate-dependent anaerobic propane and butane oxidation (n-DAPO and n-DABO) to anammox. Batch tests, isotope nitrogen experiments, multi-omics sequencing, and quantitative reverse transcription polymerase chain reaction (RT-qPCR) were jointly employed to reveal the metabolic interactions between “Ca. A. nitratireducens” and anammox bacteria, and to demonstrate the role of nitrite accumulation in stimulating the DNRA pathway of “*Ca.* A. nitratireducens”.

## Materials and methods

### Bioreactor operation

Two 1.15 L bioreactors were set-up with mixed inocula from previous enrichment cultures performing nitrate-dependent anaerobic propane or butane oxidation [10, 11] (300 mL) and enrichment cultures performing anammox (500 mL). The anammox cultures were collected from a pilot reactor (Brisbane, Australia) performing one-stage partial nitrification and anammox (PNA), and then incubated with ammonium and nitrite under anoxic conditions for 40 days. The two inocula were mixed with 120-mL anoxic synthetic medium [10], leaving a headspace of 230 mL. The bioreactors were periodically flushed with a mixed gas of 95% argon and 5% CO_2_ (Coregas, Australia) for 20 min. Pure propane or butane gas (99.99%, Coregas, Australia) were then injected into the headspace to maintain the initial 0.15–0.2 atm propane content or 0.07–0.15 atm butane. Initial nitrate and ammonium concentrations were maintained between ~5.7 and 12.9 mmol N/L by manual injection of anoxic concentrated stock solutions (80 g/L NO_3_^—^N and 46 g/L NH_4_^+^-N). In the initial 15 days, nitrite (1.1 mmol N/L) was also provided to maintain anammox activities, and then only nitrate and ammonium were added to the bioreactors. The bioreactors were operated at room temperature (22 ± 2°C) and mixed using a magnetic stirrer (IKA, Labtek, Australia) at 300 rpm. Other operational conditions are same as described previously [10]. Gas (100 μL) and liquid (0.5 mL) samples were taken regularly (four to five times a week) for the measurements of propane/butane, dinitrogen gas, nitrate, nitrite, and ammonium.

The consumption rates of propane/butane (rC_3_H_8_/rC_4_H_10_), ammonium (rNH_4_^+^), and nitrate (rNO_3_^−^) and production rate of dinitrogen gas (rN_2_-N), which were determined from the respective measured concentration profiles via linear regression, were used for mass and electron balance calculations (see Supplementary Table 1).

### Batch tests for propane bioreactor

On Day 127, a subsample of 300-mL biomass from the propane bioreactor was mixed with 180-mL medium and then transferred anaerobically to a 650-mL glass vessel for an isotope labelling experiment. The biomass was flushed with a mixed gas (95% argon and 5% CO_2_) for 20 min. Nitrate and ammonium stock solutions were added to a concentration of 6.4–7.1 mmol N/L. Pure propane gas was injected to achieve a content of ~0.15 atm. Two days after the experiment started, nitrate stock solution containing ~20% ^15^N-labelled sodium nitrate (98 atom % ^15^N, Sigma) was added to reach a total nitrate concentration of ~8.6 mmol N/L. Ammonium and propane were also added to provide sufficient electron donors. Gas and liquid sampling were conducted as described above. In addition, gas was sampled with a gas-tight syringe (Hamilton, USA) and then injected into helium-flushed vials (Exetainer, UK) for ^29^N_2_ and ^30^N_2_ determination.

The measured ^29^N_2_ and ^30^N_2_ were compared with their predicted values based on the assumption that all nitrate was reduced to nitrite by “*Ca.* A. nitratireducens”, and the produced nitrite was removed via both the anammox and denitrification reactions. The production rates of ^29^N_2_ and ^30^N_2_ were predicted as follows:

r^29^N_2_-N = (^15^N% of NO_3_^−^ × ^14^N% of NH_4_^+^ × rNH_4_^+^ + ^14^N% of NO_3_^−^ × ^15^N% of NH_4_^+^ × rNH_4_^+^) × 2.04 + ^15^N% of NO_3_^−^ × ^14^N% of NO_3_^−^ × r(NO_3_^−^)_denitrification_.

r^30^N_2_-N = ^15^N% of NO_3_^−^ × ^15^N% of NH_4_^+^ × rNH_4_^+^ × 2.04 + ^15^N% of NO_3_^−^ × ^15^N% of NO_3_^−^ × r(NO_3_^−^)_denitrification_.

r(NO_3_^−^)_denitrification_ = rNO_3_^−^ + rNH_4_^+^ × 0.26 - rNH_4_^+^ × 1.32.

where rNH_4_^+^ and rNO_3_^−^ are the measured ammonium and nitrate consumption rates, respectively, and r(NO_3_^−^)_denitrification_ represents the calculated nitrate consumption rate via full denitrification.

Batch tests were also conducted in duplicate in 160-mL serum bottles to observe nitrogen conversion when ammonium was not provided. For each bottle, 60-mL biomass from the propane bioreactor was mixed with medium (60 mL) when ammonium was nearly exhausted. The bottles were flushed with the mixed gas (95% argon and 5% CO_2_) for 20 min, and then nitrate and propane were supplied to reach ~8.6 mmol N/L and 0.15 atm, respectively. To prove anammox reaction indeed occurred in the bioreactor, several serum bottles (120 mL) were also set-up with 30-mL biomass and 30-mL medium. After flushing the bottles with the mixed gas, ammonium and nitrite were provided at 5.7–7.1 and 4.3–6.4 mmol N/L, respectively. Each batch test was conducted in triplicate.

To demonstrate if nitrite accumulation indeed triggers the DRNA pathway for “*Ca.* A. nitratireducens”, *in-situ* batch tests were conducted for the propane bioreactor with the operation divided into two stages. In Stage 1, nitrate (~2.9 mmol N/L), ammonium (~2.1 mmol N/L), and propane (~0.15 atm) were all added to the reactor, whereas in Stage 2, only nitrate and propane (the same concentrations as Stage 1) were supplied. Duplicate cycles were conducted for Stage 2. To directly observe net ammonium production from DRNA, another batch test was designed to increase activities of “*Ca.* A. nitratireducens”. This test was conducted in triplicate in 160 mL serum bottles. In this test, initial propane content was maintained ~0.15 atm in Stage 1 but increased to 0.7–0.8 atm in Stage 2, in which such a high propane concentration would increase activities of “Ca. A. nitratireducens”. Nitrate (4.4–10.1 mmol N/L) and ammonium (13.1–15.8 mmol N/L) were both provided in Stage 1 and 2. Triplicate experiments were completed. During the two batch tests, gas and liquid samples were regularly collected (three to four samples in each stage) for monitoring propane, dinitrogen gas, nitrate, nitrite, and ammonium concentrations. In addition, biomass samples (2 mL) were also collected for conducting RT-qPCR in order to compare the gene expression levels at different stages.

### Chemical analyses

The concentrations of ammonium, nitrite, and nitrate in the filtered liquid samples were measured by a flow injection analyzer (QuickChem8000, Lachat Instrument, Milwaukee, WI). Propane and nitrogen in the gas samples were quantified with a gas chromatograph (GC, 7890 A, Agilent, USA) equipped with a thermal conductivity detector [10]. Butane, ^29^N_2_, and ^30^N_2_ were determined using a GC (7890A, Agilent, USA) coupled to a quadrupole mass spectrometer (MS, 5957C inert MSD, Agilent, USA), which was operated as described previously [10].

### DNA extraction and 16S rRNA gene sequencing

Biomass samples (2 mL) were collected on Day 0 (Inoculum), 51, 105 for the propane bioreactor, and on Day 0 (Inoculum) and Day 31 for the butane reactor. The FastDNA SPIN for Soil kit (MP Biomedicals, USA) was used for DNA extraction according to the manufacture’s protocol. 16S rRNA gene (V6 to V8 regions) was amplified using the primer set 926F (5’-AAACTYAAAKGAATTGACGG-3′) and 1392R (5′- ACGGGCGGTGTGTRC-3′) and then sequenced on a NovaSeq 6000 platform (Illumina, USA) at Australian Centre for Ecogenomics, The University of Queensland (Brisbane, Australia). Sequencing results were analysed by QIIME2 as described previously [24]. The taxonomy for each feature was assigned by BLAST search against the combined non-redundant 16S and 18S SILVA database (release 138, clustered at a 99% identity) [ 25] using the classify-consensus-blast function with default parameters.

### RNA extraction and RT-qPCR

The genes *hzsA* in anammox bacteria and *nrfA* in “*Ca.* A. nitratireducens” were selected as key functional genes involved in anammox and DNRA reactions. The expression levels of these genes were quantified using RT-qPCR. RNA in the biomass samples was extracted using RNeasy Powersoil Total RNA kit (Qiagen, Germany) according to the manufacturer’s protocol, and then purified with a Turbo DNA-free kit (Thermo Fisher Scientific, USA) followed by an RNA Clean & Concentrator Kit (Zymo Research, USA). cDNA synthesis was conducted using QuantiTect Rev. Transcription Kit (Qiagen, Germany). RT-qPCR was completed by an ABI7300 system (Applied Biosystems, USA) with ChamQ SYBR Color qPCR Master Mix. Published primer sets *hzsA*_526F(TAYTTTGAAGGDGACTGG) / *hzsA*_887R (GGATAHGCRCCRTCCCAGTT) [ 26] and AMX-808-F (ARCYGTAAACGATGGGCACTAA) / AMX-1040-R (CAGCCATGCAACACCTGTRATA) [ 27] were used to amplify *hzsA* and 16S rRNA gene in anammox bacteria, respectively. Primer sets *nrfA*_1F (GCCGAGGATGAGACTGAT) / *nrfA*_2R (GAACCGAAGTGGACTTACAA) and Sym-F (CACACTGGAACTGAGACAC) / Sym-R (ACCGCTACACCTGGAATT) were designed by Primer Premier 6.0 to specifically target *nrfA* (Locus tag 01416) and 16S rRNA gene in “*Ca.* A. nitratireducens”, respectively. The specificity of the newly designed *nrfA* primer was evaluated by gel electrophoresis (Fig. S5, only a single product with a size of ~280 bp could be observed). All PCR amplifications were performed in triplicate.

### Fluorescence in situ hybridization

Biomass collected from the propane parent reactor was fixed with 4% paraformaldehyde (w/v) and stored in 1× PBS:50% ethanol (v/v) at −20°C. Fixed biomass was applied to PTFE printed slides (10 × 6 mm^2^ wells; G35–1006, ProSciTech). After drying at 37°C, biomass was dehydrated for 3 min each in 50, 80, and 96% (v/v) ethanol, again dried at 37°C, and permeabilized with lysozyme (0.05 mg/mL lysozyme in 1× PBS, 0.05 M EDTA, and 0.1 M Tris–HCl, pH 8) for 30 min at room temperature. Hybridization buffer (10 μl; 5 M NaCl, 1 M Tris/HCl, 20% formamide, 10% sodium dodecyl sulfate) was added to each sample with 1 μl of the EUB338 I–III probe mix [28], the SYMB-1018 probe targeting “*Ca.* Alkanivorans nitratireducens” [10] and the Amx820 probe targeting anaerobic ammonium oxidizing (anammox) bacteria [29]. Unlabelled helper probes of SYMB-1018 were applied in equimolar amounts [10] and the NON338 probe [30] was used as a negative hybridization control. Slides were hybridized for 3 h at 46°C, incubated in pre-heated washing buffer (5 M NaCl, 1 M Tris/Hcl, 0.5 M EDTA) for 15 min at 48°C, dipped twice for 2–3 s into ice-cold milliQ, and immediately air dried. After counterstaining with DAPI, slides were mounted with Vectashield antifade mounting medium and visualized on a Stellaris5 laser scanning confocal microscope (Leica, Germany).

### Metagenomics and metatranscriptomics

10 mL of biomass was collected from the propane bioreactor on Day 130 when the reactor showed active anaerobic ammonium and propane oxidation coupled to nitrate reduction. Biomass was preserved by adding RNAlater solution (Sigma-Aldrich) for co-extraction of total DNA and RNA. The extraction was conducted using the RNeasy Powersoil Total RNA kit with the RNeasy PowerSoil DNA Elution Kit (Qiagen, Germany) according to the manufacturer’s protocols. For metagenomic sequencing, DNA libraries were prepared using an Illumina Nextera XT DNA library preparation kit. For metatranscriptomic sequencing, DNA contamination was removed from the RNA extracts as described above. RNA library was then prepared using the TruSeq Total RNA Library Prep with Ribo-Zero Plus kit. Sequencing of both libraries was completed on a NovSeq 6000 platform using 2 × 150 bp paired end chemistry at ACE (Brisbane, Australia). The libraries generated 48 and 38 million reads for metagenomic and metatranscriptomic sequencing, respectively.

MetaWRAP [31] was used for trimming, assembly, and binning of generated reads from metagenomic sequencing. Quality of recovered genomes (completeness, contamination etc) was checked using CheckM v1.0.12 [32]. Taxonomy information of MAGs was determined by GTDB-Tk 2.1.1 [33]. Primary annotation of genomes was conducted using Prokka 1.14.5 [34]. The KEGG Orthology database and eggNOG v5 database were then searched against using kofamscan 1.3.0 and emapper 2.1.5, respectively [35, 36]. The relative abundance of each MAG was calculated using CoverM 0.6.1 (https://github.com/wwood/CoverM) with only quality primary mappings (--min-read-aligned-percent 0.95 --min-read-percent-identity 0.97).

Generated reads from metatranscriptomic sequencing were trimmed using metaWRAP “Read-QC” module [31]. Ribosomal RNA-like reads were removed using SortMeRNA 4.3.4 [37] with default settings. The relative expression of recovered MAGs was calculated similarly to the relative abundance in the metagenomic data, but mapping the metatranscriptomic reads. The expression level of each gene was quantified using featureCount [38] with the –p option. Transcripts per million [39] (TPM) were calculated to compare expression levels between genes.

### Metaproteomics

Biomass (5 mL) collected from the propane bioreactor was centrifuged (18 000 g, 4°C) and then washed with 1 × PBS for protein extraction. The extraction was conducted as detailed previously [10]. Briefly, the cell pellets were lysed using sodium dodecyl sulfate and then incubated with dithiothreitol, followed by alkylation with iodoacetamide. Protein digestion was completed in a S-Trap Micro Spin Column (ProtiFi, Huntington, USA) according to the manufacturer’s protocol. The digested peptides were analysed by liquid chromatography–tandem mass spectrometry [10]. Raw sequencing data were searched against the database built from the annotated genomes of “*Ca.* A. nitratireducens”, “Ca. Brocadia”, and “Ca. Kuenenia” in Thermo Proteome Discoverer. The identified proteins contained at least one unique peptide with a stringency cut-off of false discovery rate (FDR, *q*-value) < 0.05.

## Results

### Establishment of microbial consortia coupling anammox to n-DAPO or n-DABO

**Figure 1:**
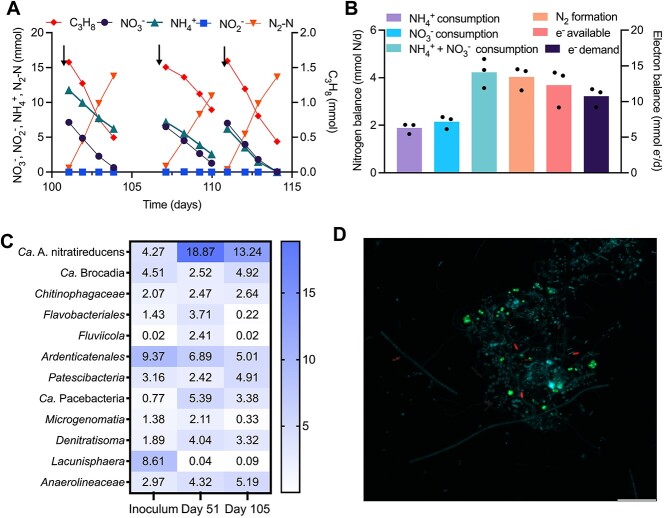
Key performance data and microbial community structure of the propane bioreactor. (A) Bioreactor performance data during the steady-state operation from Day 100 to 115. The black arrows indicate bioreactor was flushed with 95% argon and 5% CO_2_, and then nitrate, ammonium, and propane were added. Simultaneous consumption of ammonium, nitrate, and propane with dinitrogen gas production were observed. The nitrite concentration was negligible during the whole operational period. (B) Average nitrogen and electron balances calculated from reactor performance data from Day 100 to 115 (see Supplementary Table 1 for complete data and calculation). Data are presented as mean from three cycles of a single reactor in (A) and individual data points are shown by black circles. (C) Microbial community profiles at genus level in the propane bioreactor via 16S rRNA gene amplicon sequencing. Genera with an abundance of ≥2% in at least one sample are shown. (D) fluorescence micrograph of the bioreactor community hybridized with the SYMB-1018 (Cy3, red; targeting “*Ca.* A. nitratireducens”) and AMX-820 (FITC, green; targeting anammox bacteria) FISH probes and stained with DAPI (cyan; all microbial cells). The scale bar indicates 10 μm.

To prevent nitrite accumulation observed in previous enrichment bioreactors performing n-DAPO or n-DABO, two anaerobic bioreactors were set-up by mixing 300 mL of “*Ca.* A. nitratireducens” enrichment cultures [10, 11] (~20% of relative abundance) and 500 mL of anammox enrichment culture. Initially, in addition to propane/ammonium/nitrate or butane/ammonium/nitrate, the two bioreactors were also pulse-fed with nitrite to maintain anammox activities. Nitrite dosing was stopped after 15 days of operation for both reactors. Simultaneous propane/butane, ammonium, and nitrate consumption, with production of dinitrogen gas, was then observed for both reactors (Fig. 1A, Figs S1 and S2A). In contrast to previous enrichment cultures with nitrite accumulation consistently observed [10, 11], no nitrite was detected during the stable operation periods for these two reactors, indicating the effective removal of nitrite by the active anammox bacteria. In addition, propane oxidation and nitrate reduction rates in the current propane bioreactor (0.35 ± 0.11 and 2.34 ± 0.30 mmol/L/d, respectively) were ~ 17–21 times higher than our previous systems without anammox bacteria (0.02 ± 0.01 and 0.11 ± 0.04 mmol/L/d) [ 10]. Similarly, butane and nitrate consumption rates also increased from 0.09 ± 0.06 and 0.58 ± 0.30 mmol/L/d in previous systems [11] to 0.24 ± 0.10 and 2.06 ± 0.33 mmol/L/d in this butane bioreactor, respectively.

Further nitrogen balance calculations showed the total consumption rates of nitrate and ammonium in the propane (4.04 ± 0.28 mmol N/d) and butane bioreactors (3.62 ± 0.41 mmol N/d) were both close to the production rates of dinitrogen gas (4.23 ± 0.36 and 3.62 ± 0.31 mmol N/d for propane and butane reactors, respectively, Fig. 1, Fig. S2B, Supplementary Table 1). This suggests nitrate and ammonium were fully converted into dinitrogen gas as the final product. The ratios between electrons generated from ammonium and propane/butane oxidation and electrons required for nitrate reduction were 1.13 ± 0.07 and 1.13 ± 0.12 for propane and butane bioreactors, respectively (Fig. 1B, Fig. S2B, Supplementary Table 1). This indicates that nitrate was the primary electron sink for ammonium and propane/butane oxidation. Microbial community profiling with 16S rRNA gene amplicon sequencing showed that the relative abundance of “*Ca.* A. nitratireducens” increased from 4.3 to 13.2% in the propane bioreactor and from 1.3 to 5.8% in the butane bioreactor (Fig. 1C, Fig. S2C). Both the bioreactors were found to harbour the known anammox lineages of “Ca. Brocadia” (with abundances of 2.5–4.9 and 0.3–1.4% for propane and butane bioreactor, respectively) and “Ca. Kuenenia” [40, 41] (0.1–0.4 and 3.7–4.1%, Fig. 1C, Fig. S2C). In addition, fluorescence in situ hybridization images confirmed the presence of “Ca. A. nitratireducens” and anammox bacteria in the propane bioreactor (Fig. 1D). Collectively, our results support the coupling of the nitrate-dependent anaerobic propane/butane oxidation and the anammox process.

**Figure 2:**
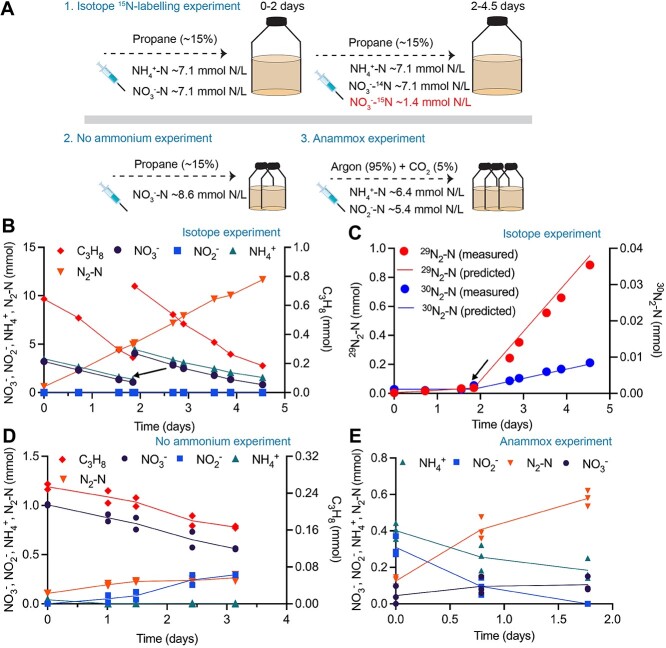
Nitrate-dependent anaerobic propane oxidation coupled to anaerobic ammonium oxidation (anammox) in the propane-fed bioreactor. (A) Experimental procedures of batch tests for propane bioreactor. The concentrations represent the initial propane, nitrate, nitrite, and ammonium concentrations. (B) Results from ^15^N-labelling batch test proving stoichiometrically balanced conversion of NO_3_^−^ and NH_4_^+^ to N_2_ without NO_2_^−^ accumulation. Propane was simultaneously consumed. (C) Measured production of ^29^N_2_ and ^30^N_2_ match predicted results based on assumed reactions (see Methods for detailed calculations). The black arrows in (B) and (C) indicate the addition of ^15^NO_3_^−^, NH_4_^+^, and propane. ^30^N_2_ was also produced due to denitrification of ^15^NO_3_^−^ and reaction between ^15^NO_2_^−^ and naturally present ^15^NH_4_^+^. (D) Batch tests showing nitrite was accumulated as a result of nitrate reduction when ammonium was not supplied. Symbols represent individual data points with the mean (*n* = 2) connected by the line. (E) Batch tests demonstrating conversion of NH_4_^+^ and NO_2_^−^ to N_2_ and NO_3_^−^ according to the anammox reaction. Symbols represent individual data points with the mean (*n* = 3) connected by the line.

To further confirm that the anammox reaction could efficiently scavenge the nitrite generated by nitrate reduction, a subsample of the propane bioreactor was incubated with unlabelled ammonium and nitrate for 2 days, and then ^15^N-labelled nitrate was added (Fig. 2A, B). After the addition of labelled nitrate, ^29^N_2_ was quickly produced as the major labelled product of dinitrogen gas (Fig. 2C). This is mainly due to the anaerobic oxidation of ^14^NH_4_^+^ with ^15^NO_2_^−^, confirming the anammox reaction removes nitrite generated from nitrate reduction. In addition, the measured production of ^29^N_2_ and ^30^N_2_ are both higher than the predicted rates if only the anammox reaction was considered, whereas they match the calculated rates by summing up the contributions of both denitrification and anammox reactions (see Methods for detailed calculations, Fig. 2C). This suggests that denitrification also contributes to nitrite reduction to dinitrogen gas (4% of total generated ^15^N-nitrite).

In batch tests without ammonium addition, nitrite accumulation and dinitrogen gas production were both observed (Fig. 2A, D), further confirming the role of anammox in nitrite removal and the contribution of denitrification to dinitrogen gas production in the parent system. Moreover, in the batch test fed ammonium and nitrite but not propane (Fig. 2A, E), the ratio between nitrite consumption/nitrate production to ammonium consumption (1.42 ± 0.05 or 0.27 ± 0.04) was close to the theoretical ratio of 1.32 or 0.26 for anammox reaction (Equation 2) [42], proving the occurrence of anammox in the propane bioreactor. Taken together, these results demonstrate nitrite generated by n-DAPO was utilized by the anammox for the generation of dinitrogen gas (Equations 1, 2, and 3): 


(1) \begin{align*} \mathrm{C}_{3}\mathrm{H}_{8}+10\mathrm{NO}_{3}^{-}\to 3\mathrm{CO}_{2}+10\mathrm{NO}_{2}^{-}+4\mathrm{H}_{2}\mathrm{O}\nonumber\\\kern7em \triangle{\mathrm{G^o}}^{{\prime}}=-1348\ \mathrm{kJ}/\mathrm{mol}\ \mathrm{C}_{3}\mathrm{H}_{8} \end{align*}

(2) \begin{align*}& \mathrm{NH}_{4}^++1.32\mathrm{N}\mathrm{O}_{2}^{-}+0.066\mathrm{HCO}_{3}^{-}+0.13\mathrm{H}^+\\\nonumber&\to 1.02\mathrm{N}_{2}+0.26\mathrm{NO}_{3}^{-}+2.03\mathrm{H}_{2}\mathrm{O}+0.066\mathrm{CH}_{2}\mathrm{O}_{0.5}\mathrm{N}_{0.15}\\\nonumber& \triangle{\mathrm{G^o}}^{{\prime}}=-358\ \mathrm{kJ}/\mathrm{mol}\ \mathrm{NH}_{4}^+ \end{align*}

(3) \begin{align*} 3\mathrm{C}_{3}\mathrm{H}_{8}+20\mathrm{N}\mathrm{O}_{2}^{-}+20\mathrm{H}^+\to 9\mathrm{CO}_{2}+10\mathrm{N}_{2}+22\mathrm{H}_{2}\mathrm{O}\nonumber\\\kern4em \triangle{\mathrm{G^o}}^{{\prime}}=-2385\ \mathrm{kJ}/\mathrm{mol}\ \mathrm{C}_{3}\mathrm{H}_{8} \end{align*}

### Syntrophic partnership between “*Ca.* A. nitratireducens” and anammox bacteria

**Figure 3:**
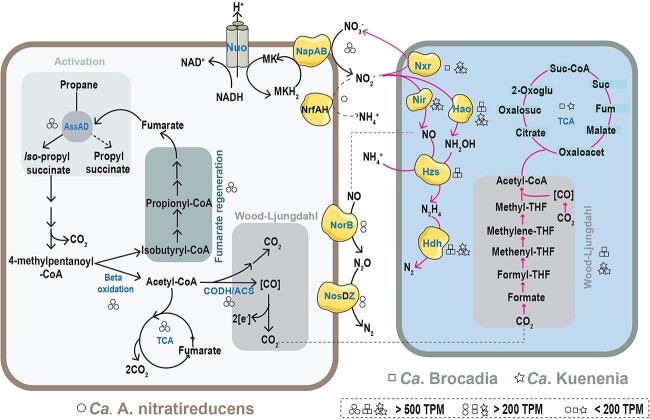
Metabolic pathways proposed for syntrophic partnership between “*Ca.* A. nitratireducens” and anammox bacteria. “Ca. A. nitratireducens” activates propane via the alkylsuccinate synthase (Ass) and finally generates CO_2_ through the oxidative tricarboxylic acid cycle or reverse Wood–Ljungdahl pathway. Nitrite generated from nitrate reduction by “Ca. A. nitratireducens” is used for ammonium oxidation to dinitrogen gas by anammox bacteria. Normalized gene expression is indicated as TPM (total transcripts per million) according to the box legend in the bottom right. Circles, squares, and star symbols represent “*Ca.* A. nitratireducens”, “Ca. Brocadia”, and “Ca. Kuenenia”, respectively. Enzymes labelled in blue text were fully or partially identified in the protein extracts. Biosynthesis and energy conservation are not indicated.

To further elucidate the partnership between “*Ca.* A. nitratireducens” and anammox bacteria, metagenomic, metatranscriptomic, and metaproteomic were applied to biomass sampled from the propane bioreactor on Day 130. Assembly and binning of the metagenomic data led to the recovery of 29 high-quality genomes (≥70% completeness and ≤ 10% contamination based on CheckM, Supplementary Data 1). These include the abundant populations of “*Ca.* A. nitratireducens”, “Ca. Brocadia”, and “Ca. Kuenenia” (11.9, 5.4, and 3.2% of relative abundance, respectively, Supplementary Table 2). Metatranscriptomic data analyses showed that these three genomes accounted for 54.4, 3.9, and 6.8% of total transcriptome reads, respectively (Supplementary Table 2), representing the most active microorganisms in the propane bioreactor. Further metabolic pathway analyses of “Ca. A. nitratireducens” suggest it encodes and expresses a full set of genes involved in nitrate reduction and anaerobic propane oxidation via fumarate addition pathway (Fig. 3, Supplementary Tables 3 and 4), including the key genes encoding nitrate reductase (*napAB*) and alkylsuccinate synthase (*assAD*). Most of these gene products were also detected in protein extracts (Supplementary Tables 3 and 4), indicating “Ca. A. nitratireducens” is responsible for the observed nitrate-dependent anaerobic propane oxidation. Genes encoding nitric oxide reductase (*norB*) and nitrous oxide reductase (*nosZ*) were also expressed and identified in protein extracts (Fig. 3, Supplementary Table 4), consistent with the performance data that denitrification contributed to dinitrogen gas production in the bioreactor. These observations demonstrate “Ca. A. nitratireducens” is able to reduce nitrate to dinitrogen gas, although the nitrite reduction step is unclear, consistent with our previous system without anammox bacteria [10, 11]. Moreover, genes involved in key anammox metabolisms, including hydrazine synthase (*hzsA*), hydrazine dehydrogenase (*hdh*), hydroxylamine oxidoreductase (*hao*), nitrite reductase (*nirS*), and nitrite oxidoreductase (*nxr*), were highly expressed in “Ca. Brocadia” and “Ca. Kuenenia”, together with the gene products detected by metaproteomic analyses (Fig. 3, Supplementary Table 5). The Wood–Ljungdahl pathways of both “Ca. Brocadia” and “Ca. Kuenenia” were also highly expressed (Supplementary Data 2), suggesting the carbon dioxide generated from propane oxidation was likely utilized by anammox bacteria for autotrophic growth (Fig. 3). A search of the entire metagenome library confirmed “Ca. Brocadia” and “Ca. Kuenenia” as the only bacteria containing *hzsA* and *hdh* genes, indicating other bacteria in the microbial community unlikely play a direct role in anammox. These results confirm anammox bacteria play a major role in removing nitrite and generating dinitrogen gas, and also likely supply “Ca. A. nitratireducens” with nitrate in the propane bioreactor. Collectively, our data provide compelling evidence that “Ca. A. nitratireducens” and anammox populations (consisting of “Ca. Brocadia” and “Ca. Kuenenia”) can form a syntrophic partnership (Fig. 3), in which nitrite produced by “Ca. A. nitratireducens” is used as an electron acceptor for oxidizing ammonium to dinitrogen gas by anammox bacteria.

### Nitrate depletion and nitrite accumulation triggers DNRA process by “*Ca.* A. nitratireducens”

In previous enrichment cultures without abundant anammox bacteria, the DNRA process was consistently observed when nitrate was depleted and nitrite was accumulated [10, 11]. Cytochrome *c* nitrite reductase in “*Ca*. A. nitratireducens” (*nrfA*, Locus tag 01415) responsible for DNRA was also highly expressed with its protein products detected during this process [10, 11]. In contrast, ammonium production was not observed in the current bioreactors when nitrite accumulation was prevented by the activity of the anammox bacteria (Fig. 1A, S1A). Moreover, *nrfA* expression was low and not detected in protein extracts (Supplementary Table 4), confirming the minimal activity of the DNRA pathway in current system where anammox scavenge nitrite. These results indicate that nitrite accumulation is likely a key factor in triggering DNRA mediated by “*Ca*. A. nitratireducens”.

To further support this hypothesis, batch tests were conducted and analysed at points where supplied ammonium was present (Stage 1) and when it had become exhausted (Stage 2) for the propane bioreactor. Nitrite accumulated (to 0.9–2.4 mmol N/L) after supplied ammonium was exhausted (Stage 2; Fig. S3A). As only trace ammonium production was observed during Stage 2, we suspect that any generated ammonium from DNRA was immediately consumed by anammox bacteria present. This was confirmed by RT-qPCR results, which showed the expression levels of *nrfA* on Days 2 and 4 (where nitrate was completely consumed and nitrite had accumulated) were 19.0- and 5.3-fold higher than when ammonium was still present (Stage 1; Fig. S3B), respectively. In addition, the *hzsA* transcription levels at these Stage 2 time points are comparable with Stage 1, suggesting ammonium oxidation likely occurred during the nitrite accumulation periods. Therefore, DNRA and ammonium oxidation likely simultaneously occurred in Stage 2 when nitrate was depleted and nitrite accumulated, leading to the utilization of ammonium before it can accumulate.

**Figure 4:**
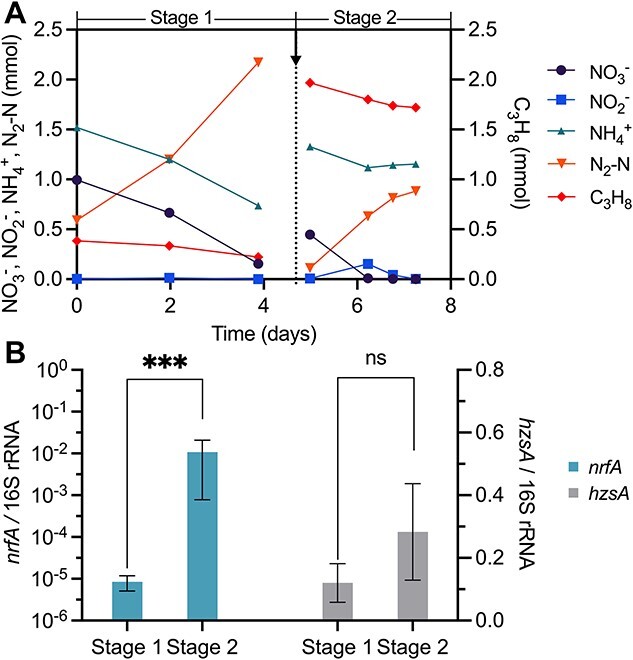
Nitrate depletion and nitrite accumulation trigger DNRA process in the propane bioreactor. (A) No nitrite accumulation or ammonium production was observed in Stage 1 when ~0.15 atm propane was supplied, whereas nitrite was accumulated with ammonium production when the propane partial pressure was increased to ~0.7–0.8 atm in Stage 2. The black arrow indicates the bioreactor was flushed with argon and CO_2_, and the addition of propane, nitrate, and ammonium. Symbols represent the mean (n = 3). (B) RT-qPCR results showing the expression of *nrfA* in “*Ca.* A. nitratireducens” was significantly upregulated (***P < 0.001) whereas there were no significant differences (ns) for *hzsA* in anammox bacteria in Stage 2 compared to Stage 1. The expression levels of *nrfA* and *hzsA* were normalized to the expression levels of 16S rRNA gene of “Ca. A. nitratireducens” and anammox bacteria, respectively. Error bars indicate standard deviations of samples from three tests.

To directly observe ammonium production by the DNRA process, subcultures from the propane parent reactor were incubated with a headspace propane partial pressure of ~0.15 atm (Stage 1) in 160-mL serum bottles and then propane content was increased to ~0.7–0.8 atm (Stage 2). The increased propane content in Stage 2 was performed due to the increased activities of “*Ca.* A. nitratireducens” under higher propane concentrations. Indeed, nitrate reduction rates increased from 0.22 ± 0.01 mmol/d in Stage 1 to 0.35 ± 0.01 mmol/d in Stage 2, resulting in nitrite accumulated to 1.4–1.7 mmol N/L (Fig. 4A). Net ammonium production was then observed at the end of each cycle in Stage 2 when nitrate was depleted and high levels nitrite had accumulated (Fig. 4A). In accordance with the occurrence of DNRA in Stage 2, the *nrfA* expression was significantly upregulated in Stage 2 when nitrate was depleted and nitrite was accumulated (Fig. 4B). These results collectively support the roles of nitrate depletion and high nitrite concentrations in triggering DNRA by “*Ca*. A. nitratireducens”.

## Discussion

This work demonstrated a sustained syntrophic partnership between anammox bacteria and the SCGA oxidizing “*Ca.* A. nitroreducens”. In this partnership, “*Ca*. A. nitroreducens” was found to anaerobically oxidize propane or butane coupled to nitrate reduction, with generated nitrite used by anammox for the oxidation of ammonium. The benefit of the partnership to “*Ca.* A. nitratireducens” was evident from the significant increase in rates for SCGA oxidation and nitrate reduction. The enhanced activities of “Ca. A. nitratireducens” are attributed to the anammox bacteria scavenging nitrite generated by nitrate reduction, thus eliminating the inhibitory effect of nitrite accumulation on the SCGA oxidizer. Therefore, the development of efficient bioreactors for nitrate-dependent anaerobic SCGA oxidation should consider the incorporation of nitrite scavengers such as anammox bacteria. This multispecies interaction may also be applied for SCGAs-based simultaneous nitrate and ammonium removal from contaminated groundwater [43, 44].

In contrast to our previous “*Ca*. A. nitratireducens” enrichments propagated under high propane/butane concentrations (~0.9–1.1 atm), the lower levels of SCGAs (~0.05–0.2 atm) promoting the partnership between “Ca. A. nitratireducens” and anammox bacteria in this study are much closer to the SCGA concentrations in natural environments (up to 0.1 atm) [ 6, 45, 46]. This work indicates the potential for syntrophic partnerships between “Ca. A. nitratireducens” and nitrite scavengers like anammox in natural ecosystems. Indeed, anammox bacteria are widely found in marine and terrestrial ecosystems, including hydrocarbon-rich seeps, hydrothermal vent sediments, and oil-contaminated sites [47–49], where SCGAs and nitrogen are available. Therefore, the interactions between anammox bacteria and “Ca. A. nitratireducens” could potentially occur in these SCGA-rich hotspots. Further studies are required to investigate the global distribution of their co-occurrence and confirm their contributions to the cycling of SCGAs and nitrogen compounds. Given the co-presence of “Ca. A. nitratireducens” and anammox gave substantially higher propane and butane consumption rates (up to 17 times higher), such syntrophic partnerships may have a significant impact on the mitigation of atmospheric SCGA emissions in the environment.

**Figure 5:**
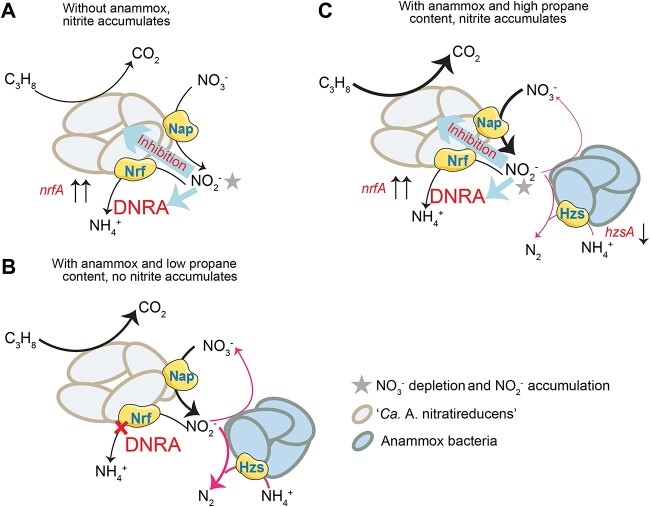
Responses of “*Ca.* A. nitratireducens” under various scenarios with and without occurrence of DNRA in the propane-fed bioreactor. (A) In the absence of anammox bacteria, consistent nitrite accumulation triggers DNRA. (B) Anammox process avoids nitrite being accumulated under the condition of low propane content (~0.05–0.2 atm), thus suppressing DNRA. (c) Inhibition of anammox activities and increase of “Ca. A. nitratireducens” activities by high propane content (>0.6 atm) lead to nitrite accumulation and thus reoccurrence of DNRA. Nitrite accumulation in scenarios (A) and (C) inhibits the metabolisms of “*Ca*. A. nitratireducens”, thus stimulating expression of DNRA pathways, as evidenced by ammonium production and upregulation of *nrfA*.

This study provides evidence that nitrate depletion and nitrite accumulation are key factors stimulating expression of DNRA pathways for “*Ca*. A. nitratireducens”. In the absence of the anammox process in previous enrichment studies, the lack of *nirS/K* in “*Ca*. A. nitratireducens” genome is likely responsible for nitrite being accumulated. Subsequently, the DNRA pathway was upregulated in response to oxidative stress of nitrite on the cell (Fig. 5A). With anammox bacteria effectively scavenging nitrite produced by “Ca. A. nitratireducens” in this study, nitrite inhibition is alleviated, thus suppressing the DNRA process (Fig. 5B). However, high propane content could lead to an increase in nitrate reduction and a decrease in ammonium oxidation rates, leading to nitrite accumulation and subsequent DNRA (Fig. 5C). In addition, DNRA appeared to occur when nitrate was completely consumed (Fig. 4A), indicating the depletion of nitrate also plays a role in triggering DNRA (Fig. 5). This is likely because “Ca. A. nitratireducens” switch to DNRA in the absence of nitrate as their preferrable electron acceptor. In summary, nitrate depletion and nitrite accumulation both appear to be key factors triggering DNRA by “Ca. A. nitratireducens”. Given “Ca. A. nitratireducens” cannot use nitrite as their direct electron acceptor (Fig. S4), it is assumed that DNRA is only used as a temporary metabolic strategy to deal with nitrite stress, but unable to support continuous growth of “Ca. A. nitratireducens”. High nitrite concentrations are also known to trigger DNRA for multiple microorganisms such as the anaerobic methane oxidizing archaeon “*Ca.* M. nitroreducens” and *Bacillus vireti*, *Aeromonas* sp., *Citrobacter* sp., and *Shewanella* sp. [50–52]. These microorganisms all lack *nirS/K*, thus relying on DNRA to remove accumulated nitrite. In addition, high nitrite to nitrate ratios also favours DNRA over denitrification for *Shewanella loihica* strain PV-4 which harbours all the genes for these two reduction pathways [53]. These observations support DNRA as an important metabolic strategy to remove nitrite to alleviate oxidative stress. Considering high nitrite is frequently detected in terrestrial and aquatic ecosystems [54], this phenomenon is likely common in natural ecosystems. In contrast to denitrification generating gaseous N_2_ as the final product, DNRA produces ammonium to retain nitrogen in the system. As such, the findings of this study have important implications for our understanding of the fate of nitrogen and SCGAs in anaerobic environments.

## Supplementary Material

Supporting_info-C3AX_wrae063

Supplementary_data_1-MAG_info-revised_wrae063

Supplementary_data_2_Anammox_metabolism_wrae063

## Data Availability

Metagenomic, metatranscriptomic raw sequencing data, and the MAGs are archived in NCBI database under Project number PRJNA984423. The mass spectrometry proteomics data have been deposited to the ProteomeXchange Consortium via the PRIDE partner repository with the dataset identifier PXD039266.
