# Supplementary material for: Anaerobic oxidation of ammonium and short-chain gaseous alkanes coupled to nitrate reduction by a bacterial consortium
Source: ISME J. 2024 Apr 16;18(1):wrae063. doi: 10.1093/ismejo/wrae063 (PMC11090206; doi:10.1093/ismejo/wrae063)
Supplement: Supporting_info-C3AX_wrae063 [file supporting_info-c3ax_wrae063.docx]

**Anaerobic oxidation of ammonium and short-chain gaseous alkanes coupled to nitrate reduction by a bacterial consortium**

**Short title: Anammox boost NO_3_^-^-driven SCGA oxidation**

Mengxiong Wu^1^, Xiawei Liu^1^, J. Pamela Engelberts^2^, Gene W. Tyson^2^, Simon J. McIlroy^2^, Jianhua Guo^1,^ *

^1^Australian Centre for Water and Environmental Biotechnology (ACWEB, formerly AWMC), The University of Queensland, St Lucia, Queensland, Australia.

^2^Centre for Microbiome Research, School of Biomedical Sciences, Queensland University of Technology (QUT), Translational Research Institute, Woolloongabba, Queensland, Australia

*Corresponding author email: [jianhua.guo@uq.edu.au](mailto:jianhua.guo@uq.edu.au)

**
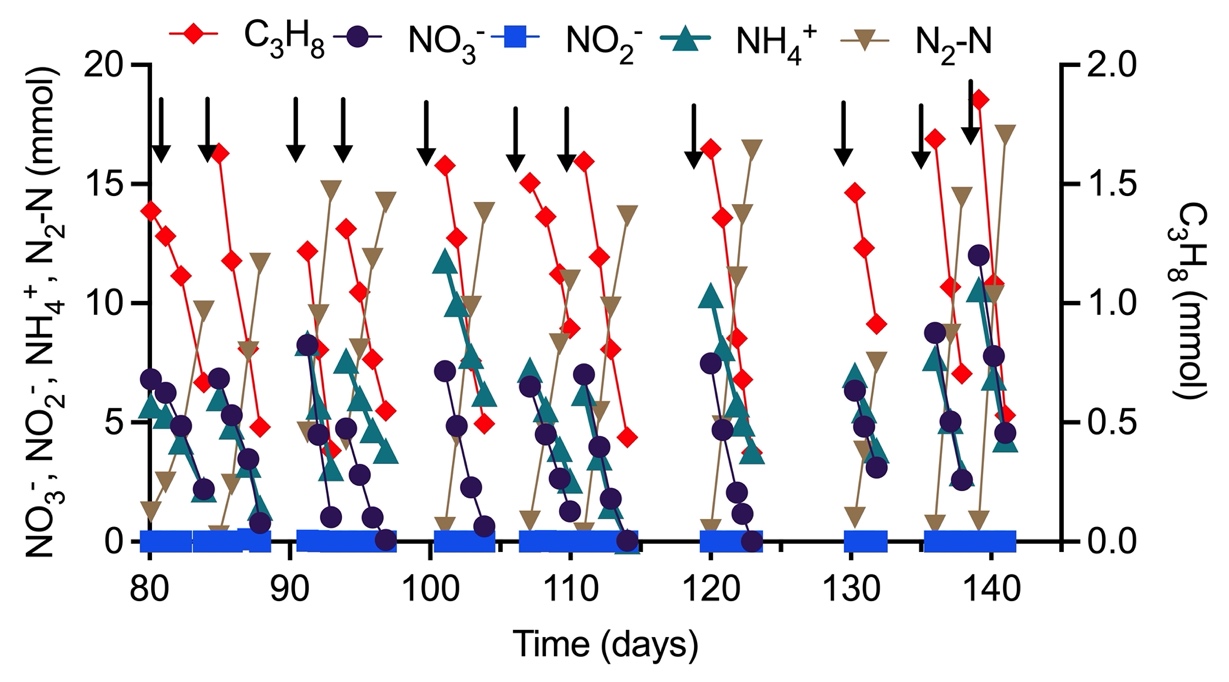
**

**Fig. S1 Long-term performance of the propane-fed bioreactor.** Consistent propane, ammonium and nitrate consumption with production of dinitrogen gas were observed. The black arrows indicate bioreactor was flushed with 95% argon and 5% CO_2_, and then nitrate, ammonium and propane were added.

**
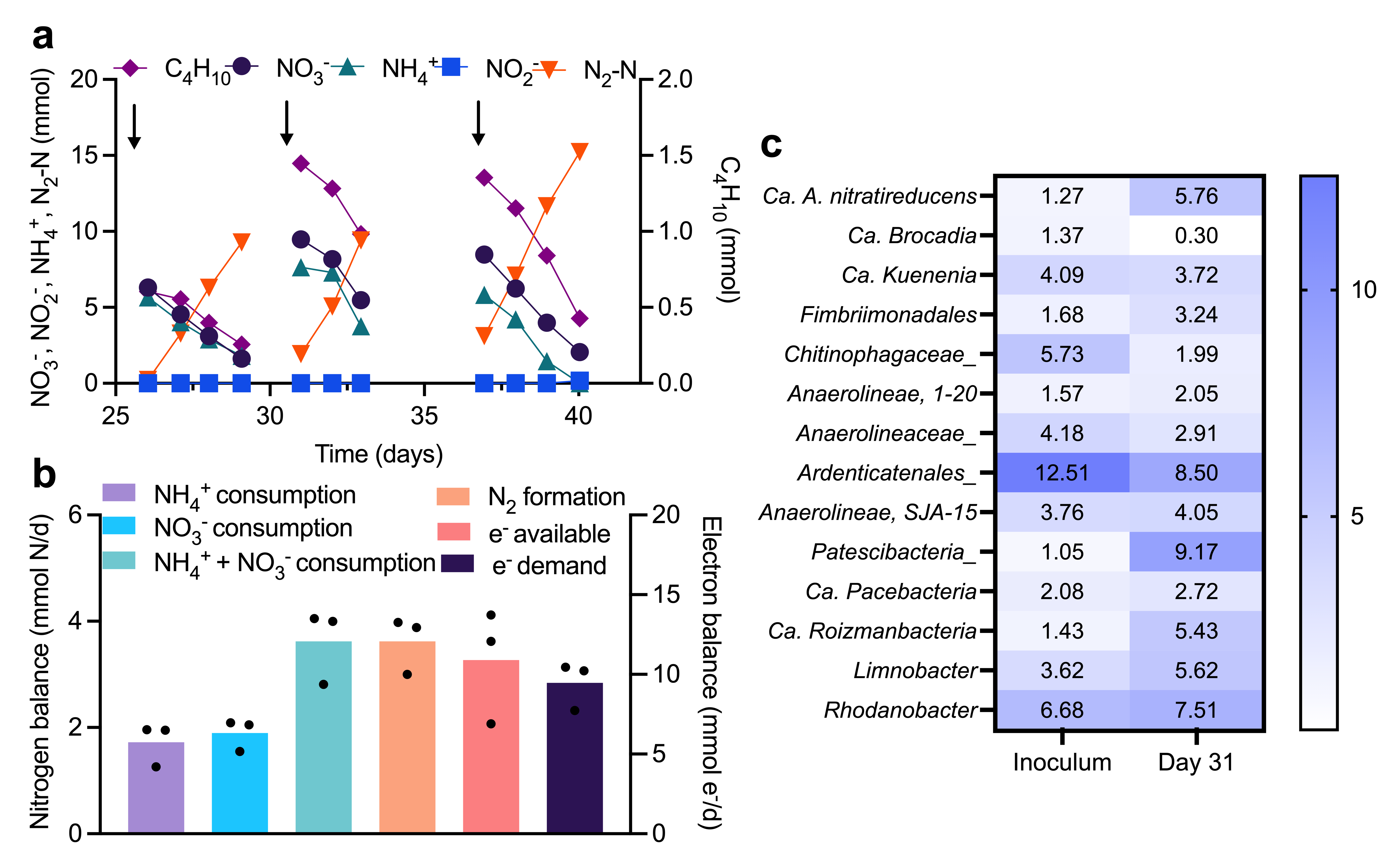
**

**Fig. S2 Key performance data and microbial community structure of the butane-fed bioreactor. a,** Bioreactor performance data during stable-stage operation from Day 25 to 40. The black arrows indicate bioreactor flushing with 95% argon and 5% CO_2_, and addition of nitrate, ammonium and butane. Simultaneous consumption of ammonium, nitrate and butane with dinitrogen gas production were observed. The nitrite concentration was negligible during the whole operational period. **b**, Average nitrogen and electron balances calculated from reactor performance data from Day 25 to 40 (see Supplementary Table 1 for complete data and calculation). Data are presented as mean from three cycles of a single reactor in **a** and individual data points are shown by black circles. **c,** Microbial community profiles at genus level in butane bioreactor via 16S rRNA gene amplicon sequencing. Genera with an abundance of ≥2% in at least one sample are shown. Populations not resolved at the genus level are indicated with an underscore.

**
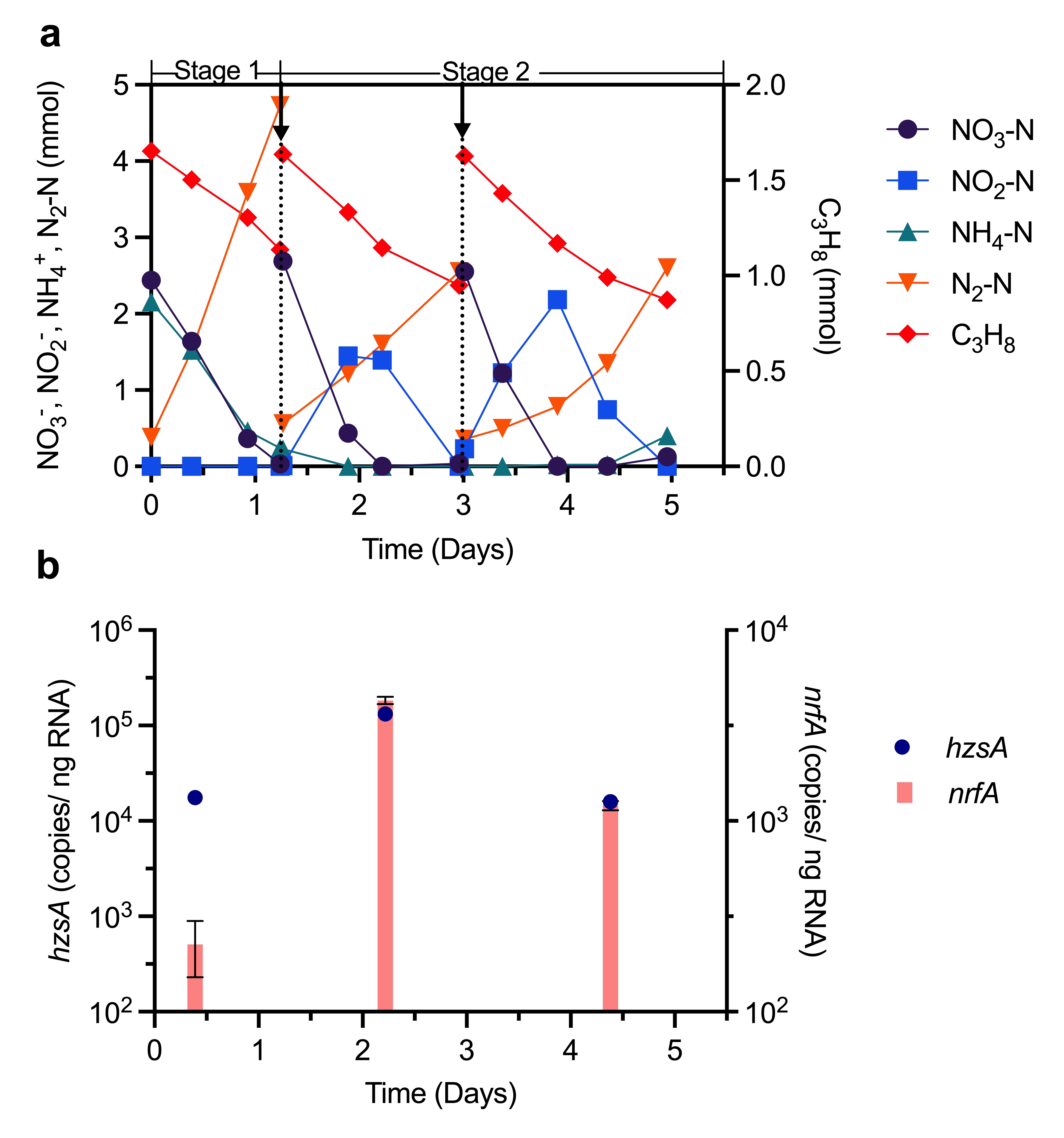
**

**Fig. S3 Batch test and RT-qPCR results for the propane-fed bioreactor. a,** No nitrite accumulation was observed when ammonium was provided in Stage 1, while high nitrite accumulation was observed in Stage 2 after ammonium was depleted. The black arrows indicates when the reactor was flushed with argon, and propane and nitrate were added. **b,** transcription profiles of *hzsA* in anammox bacteria and *nrfA* in ‘*Ca*. A. nitratireducens’ during the batch test. Error bars represent standard deviations of technical triplicates. The absence of visible bars indicates that the error is smaller than the symbol size.


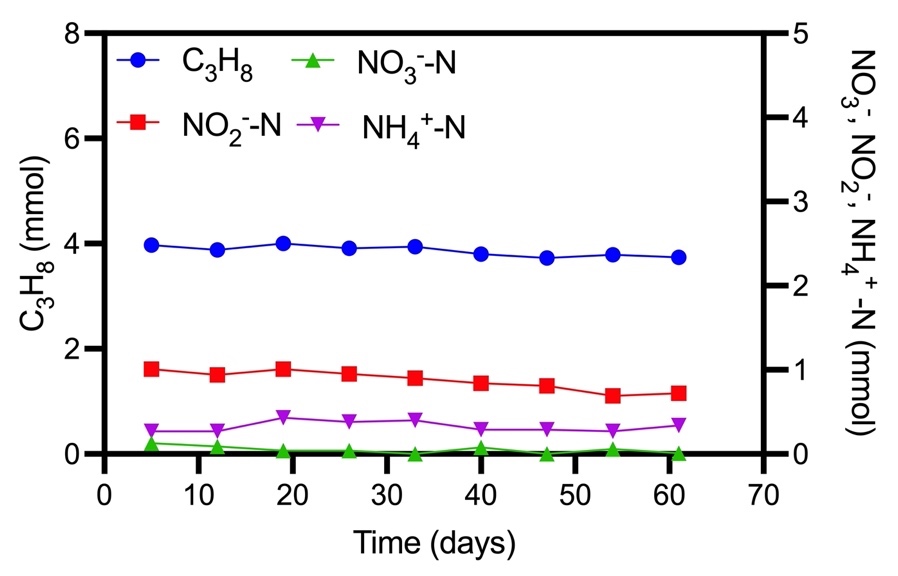


**Fig. S4** Incubation of previous enrichment cultures (only ‘*Ca*. A. nitratireducens’, without anammox bacteria) with only nitrite as the electron acceptor. Results showed that no obvious propane consumption, nitrite reduction or ammonium production were observed, indicating ‘*Ca*. A. nitratireducens’ cannot use nitrite as their direct electron acceptor.


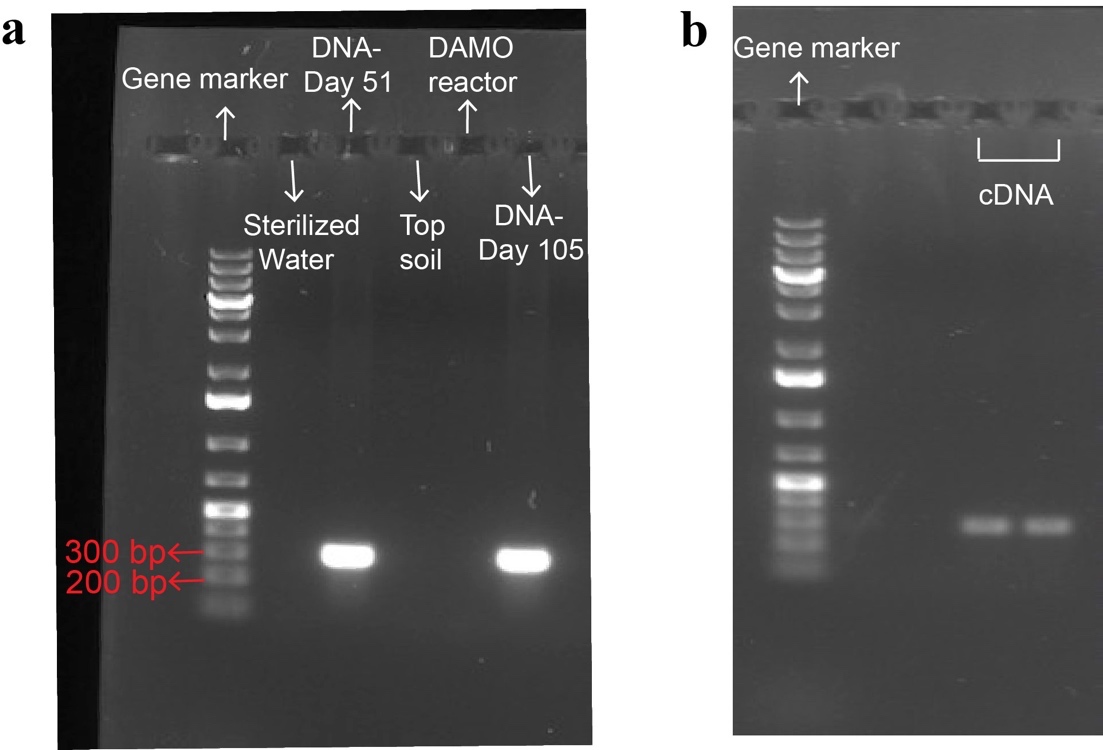


**Fig. S5** **Gel analyses for PCR products of *nrfA* in ‘*Ca*. A. nitratireducens’ using custom primers. a,** Only a single band can be observed for DNA extracted from biomass samples (Day 51 and Day 105) collected from the propane-fed bioreactor, while sterilized water, and DNA from a topsoil sample and a denitrifying anaerobic methane oxidation (DAMO) reactor all show negative results. **b,** cDNA synthesized from RNA samples of the propane-fed bioreactor also show a single band with the same size as DNA.

**Supplementary Table 1** Nitrogen and electron balance calculation based on propane and butane bioreactor performance data collected from Day 100 to 115 and Day 25 to 40, respectively.

| Nitrogen and electron balance (mmol/d) | rC_3_H_8_ / rC_4_H_10_ oxidized | rNO_3_^-^reduced | rNH_4_^+^ oxidized | rN_2_-N generated | Electrons generated by C_3_H_8_ / C_4_H_10_ and NH_4_^+^ oxidation^1^ | Electrons demand for NO_3_^-^reduction^2^ | Nitrogen balance^3^ | Electron balance^4^ |
| --- | --- | --- | --- | --- | --- | --- | --- | --- |
| Propane reactor | 0.40 | 2.35 | 2.01 | 4.79 | 14.07 | 11.74 | 1.10 | 1.20 |
|  | 0.21 | 1.84 | 1.63 | 3.57 | 9.18 | 9.21 | 1.03 | 1.00 |
|  | 0.38 | 2.26 | 2.02 | 4.33 | 13.63 | 11.3 | 1.01 | 1.21 |
| Butane reactor | 0.12 | 1.55 | 1.26 | 3.00 | 6.90 | 7.73 | 1.07 | 0.89 |
|  | 0.24 | 2.05 | 1.95 | 3.88 | 12.08 | 10.23 | 0.97 | 1.18 |
|  | 0.30 | 2.09 | 1.96 | 3.98 | 13.72 | 10.45 | 0.98 | 1.31 |

^1^Electrons generated by C_3_H_8_ / C_4_H_10_ and NH_4_^+^ oxidation = rC_3_H_8_ $\times$ 20 or rC_4_H_10_ $\times$26 + rNH_4_^+^ $\times$ 3.

^2^Electrons demand for NO_3_^-^ reduction = rNO_3_^-^reduced $\times$ 5.

^3^Nitrogen balance = rN_2_-N generated / (rNO_3_^-^reduced + rNH_4_^+^ oxidized).

^4^Electrons generated by C_3_H_8_ / C_4_H_10_ and NH_4_^+^ oxidation divided by electrons demand for NO_3_^-^ reduction; theoretically higher than 1.0, due to a fraction of carbon assimilated into biomass cells.

**Supplementary Table 2** Relative abundance and expression profiles of high-quality MAGs analysed by metagenomic and metatranscriptomic sequencing (≥ 1% relative abundance are shown). Abundance and expression of each genome were calculated using CoverM 0.6.1 (<https://github.com/wwood/CoverM>) with only quality primary mappings.

| **Relative abundance**  **(%)** | **Relative expression (%)** | **GTDB classification** |
| --- | --- | --- |
| 11.93 | 54.43 | d__Bacteria;p__Firmicutes_E;c__Symbiobacteriia;o__;f__;g__;s__ |
| 5.43 | 3.93 | d__Bacteria;p__Planctomycetota;c__Brocadiae;o__Brocadiales;f__Brocadiaceae;g__Brocadia;s__Brocadia sapporoensis |
| 3.22 | 6.81 | d__Bacteria;p__Planctomycetota;c__Brocadiae;o__Brocadiales;f__Brocadiaceae;g__Kuenenia;s__Kuenenia stuttgartiensis |
| 12.55 | 3.44 | d__Bacteria;p__Chloroflexota;c__Anaerolineae;o__SSC4;f__SSC4;g__SSC4;s__SSC4 sp013693795 |
| 2.71 | 0.45 | d__Bacteria;p__KSB1;c__UBA2214;o__DRLW01;f__QEVD01;g__QEVD01;s__QEVD01 sp013359425 |
| 2.44 | 2.40 | d__Bacteria;p__Armatimonadota;c__Chthonomonadetes;o__Chthonomonadales;f__CAJBBX01;g__JABWBA01;s__JABWBA01 sp013360745 |
| 2.14 | 0.16 | d__Bacteria;p__Proteobacteria;c__Gammaproteobacteria;o__Burkholderiales;f__Burkholderiaceae;g__SCN-69-89;s__ |
| 2.05 | 0.45 | d__Bacteria;p__Chloroflexota;c__Anaerolineae;o__Anaerolineales;f__EnvOPS12;g__UBA7227;s__UBA7227 sp002473085 |
| 1.88 | 0.03 | d__Bacteria;p__Proteobacteria;c__Alphaproteobacteria;o__Rhizobiales_A;f__Rhizobiaceae_A;g__;s__ |
| 1.80 | 0.74 | d__Bacteria;p__Acidobacteriota;c__Holophagae;o__Acanthopleuribacterales;f__;g__;s__ |
| 1.77 | 0.08 | d__Bacteria;p__Verrucomicrobiota;c__Kiritimatiellae;o__LD1-PB3;f__CAIVKH01;g__;s__ |
| 1.63 | 0.71 | d__Bacteria;p__Bacteroidota;c__Bacteroidia;o__Chitinophagales;f__Saprospiraceae;g__UBA3362;s__UBA3362 sp019187365 |
| 1.51 | 3.02 | d__Bacteria;p__Acidobacteriota;c__Blastocatellia;o__Pyrinomonadales;f__Pyrinomonadaceae;g__OLB17;s__OLB17 sp015075585 |
| 1.45 | 0.51 | d__Bacteria;p__Planctomycetota;c__Phycisphaerae;o__Phycisphaerales;f__Phycisphaeraceae;g__;s__ |
| 1.12 | 0.45 | d__Bacteria;p__Bacteroidota;c__Ignavibacteria;o__Ignavibacteriales;f__Ignavibacteriaceae;g__H2-BAC3;s__ |
| 1.14 | 0.11 | d__Bacteria;p__Bacteroidota;c__Bacteroidia;o__Chitinophagales;f__Chitinophagaceae;g__UTBCD1;s__ |

**Supplementary Table 3** Genes encoding enzymes involved in anaerobic propane oxidation in ‘*Ca.* A. nitratireducens’. The TPM expression values of *Ca.* A. nitratireducens’ in the reactor of this study were compared with Stage 1 and 2 of previous study.

| **Gene** | **Feature of gene product** | | **Locus Tag** | **EC**  **number** | **KO number** | **Transcriptome**  **(TPM)** | **Log2 ratio of TPM expression values** | | **Proteome**  **(Unique peptides)** |
| --- | --- | --- | --- | --- | --- | --- | --- | --- | --- |
|  |  |  |  |  |  |  | **This reactor/**  **Stage 1** | **This reactor/**  **Stage 2** |  |
| **Propane activation and methylmalonyl-CoA pathway** | | | | | | | | | |
| *assA1* | Alkylsuccinate synthase alpha subunit | | 00048 | 4.1.99.11 | K07540 | 882.8 | 3.34 | 2.16 | 2 |
| *assD1* | Alkylsuccinate synthase activating protein | | 00050 | 1.97.1.- | K20037 | 2700.6 | 3.32 | 2.75 | 2 |
| *assA2* | Alkylsuccinate synthase alpha subunit | | 00051 | 4.1.99.11 | K07540 | 65114.2 | 3.49 | 1.63 |  |
| *assD2* | Alkylsuccinate synthase activating protein | | 00053 | 1.97.1.- | K20037 | 2792.4 | 2.66 | 2.92 | 2 |
| *assA3* | Alkylsuccinate synthase alpha subunit | | 00054 | 4.1.99.11 | K07540 | 64204.9 | 2.47 | 1.93 | 9 |
| *fadD* | Long-chain acyl-CoA synthetase | | 02045 | 6.2.1.3 | K01897 | 135.5 | 1.40 | 1.25 | 1 |
| *mcmA1* | Methylmalonyl-CoA mutase, N-terminal domain | | 01249 | 5.4.99.2 | K01848 | 726.9 | 0.81 | 1.35 | 4 |
| *mcmA2* | Methylmalonyl-CoA mutase, C-terminal domain | | 01250 | 5.4.99.2 | K01849 | 693.9 | 0.36 | 0.89 | 2 |
| *mcmA3* | Methylmalonyl-CoA mutase, N-terminal domain | | 02037 | 5.4.99.2 | K01848 | 809.3 | 0.48 | 1.27 | 3 |
| *mcmA4* | Methylmalonyl-CoA mutase, C-terminal domain | | 02038 | 5.4.99.2 | K01849 | 710.3 | 0.32 | 0.99 |  |
| *mcmA5* | Methylmalonyl-CoA mutase, N-terminal domain | | 01858 | 5.4.99.2 | K01848 | 303.1 | 0.06 | 0.16 |  |
| *pccB1* | Propionyl-CoA carboxylase beta chain | | 01254 | 6.4.1.3 | K01965 | 204.8 | 0.14 | 0.64 |  |
| *pccB2* | Propionyl-CoA carboxylase beta chain | | 01261 | 6.4.1.3 | K01965 | 282.8 | 0.04 | 0.75 |  |
| *pccB3* | Propionyl-CoA carboxylase beta chain | | 01861 | 6.4.1.3 | K01965 | 203.6 | 1.49 | 0.69 |  |
| *pccB4* | Probable Propionyl-CoA carboxylase beta chain | | 02460 | 6.4.1.3 | K01965 | 183.3 | 0.86 | 2.46 | 2 |
| **Beta oxidation** | | | | | | | | | |
| *eftA1* | Electron transfer flavoprotein alpha subunit | | 00756 | 1.3.1.108 | K22432 | 880.5 | 0.49 | 1.97 | 4 |
| *eftB1* | Electron transfer flavoprotein beta subunit | | 00757 | 1.3.1.108 | K22431 | 847.6 | 0.44 | 1.86 | 1 |
| *eftB2* | Electron transfer flavoprotein beta subunit | | 02282 | 1.3.1.108 | K22431 | 14.9 | 2.76 | / |  |
| *eftA2* | Electron transfer flavoprotein alpha subunit | | 02283 | 1.3.1.108 | K22432 | 10.8 | 0.30 | 1.08 |  |
| *fadA1* | Acetyl-CoA acyltransferase | | 00777 | 2.3.1.9 | K00626 | 113.9 | 0.89 | 1.28 |  |
| *fadA2* | Acetyl-CoA acyltransferase | | 01243 | 2.3.1.9 | K00626 | 201.7 | 0.58 | 0.65 |  |
| *fadA3* | Acetyl-CoA acyltransferase | | 01258 | 2.3.1.9 | K00626 | 453.5 | -0.37 | -0.64 | 1 |
| *fadA4* | Acetyl-CoA acyltransferase | | 01406 | 2.3.1.9 | K00626 | 412.4 | -0.12 | 1.39 | 2 |
| *fadA5* | Acetyl-CoA acyltransferase | | 01945 | 2.3.1.16 | K00632 | 145.6 | / | 0.35 |  |
|  | Acyl-CoA transferase | | 01259 |  |  | 386.1 | -0.23 | -0.42 |  |
| *fadB1* | Acyl-CoA dehydrogenase | | 00775 | 1.3.8.7 | K00249 | 115.1 | 0.62 | 1.07 |  |
| *fadB2* | Acyl-CoA dehydrogenase | | 01246 | 1.3.8.7 | K00249 | 534.2 | 0.44 | 0.96 | 2 |
| *fadB3* | Acyl-CoA dehydrogenase | | 01260 | 1.3.8.7 | K00249 | 305.9 | 0.23 | 0.85 |  |
| *fadB4* | Acyl-CoA dehydrogenase | | 01944 | 1.3.8.7 | K00249 | 112.4 | 0.14 | 0.53 | 1 |
| *fadB5* | Acyl-CoA dehydrogenase | | 02040 | 1.3.8.7 | K00249 | 1109.6 | 0.01 | 1.19 |  |
| *echA1* | Enoyl-CoA hydratase/isomerase | | 00187 | 4.2.1.17 | K01715 | 683.7 | -0.04 | 0.56 |  |
| *echA2* | Enoyl-CoA hydratase/isomerase | | 00760 | 4.2.1.17 | K01692 | 39.9 | 0.50 | -0.31 |  |
| *echA3* | Enoyl-CoA hydratase/isomerase | | 01255 | 4.2.1.17 | K01692 | 279.4 | -0.31 | 0.14 |  |
| *echA4* | Enoyl-CoA hydratase/isomerase | | 02356 | 4.2.1.17 | K01692 | 293.6 | 0.08 | 0.97 |  |
|  | 3-hydroxyacyl-CoA dehydrogenase (NAD binding domain) | | 01407 | 1.1.1.157 | K00074 | 575.1 | -0.30 | 0.27 | 1 |
|  |  | **TCA cycle** | | | | | | | |
|  | Citrate (re)-synthase | | 01713 | 2.3.3.3 | K05942 | 1408.6 | -0.45 | -0.21 | 6 |
|  | Aconitate hydratase | | 02416 | 4.2.1.3 | K01681 | 374.7 | / | / | 3 |
| *icd* | Isocitrate dehydrogenase [NADP] | | 01714 | 1.1.1.42 | K00031 | 930.2 | -0.14 | 1.01 | 6 |
| *oorD* | 2-oxoglutarate ferredoxin oxidoreductase subunit delta | | 01853 | 1.2.7.3 | K00176 | 1634.0 | -0.06 | 0.16 |  |
| *oorA* | 2-oxoglutarate ferredoxin oxidoreductase subunit alpha | | 01854 | 1.2.7.3 | K00174 | 1485.1 | 0.25 | 0.64 | 3 |
| *oorB* | 2-oxoglutarate ferredoxin oxidoreductase subunit beta | | 01855 | 1.2.7.3 | K00175 | 2046.2 | 0.34 | 0.80 | 3 |
| *oorC* | 2-oxoglutarate ferredoxin oxidoreductase subunit gamma | | 01856 | 1.2.7.3 | K00177 | 1461.4 | 0.09 | 0.47 |  |
| *sucD* | Succinate-CoA ligase (ADP-forming) beta subunit | | 02013 | 6.2.1.5 | K01903 | 1320.5 | -0.34 | 1.45 | 5 |
| *sucC* | Succinate-CoA ligase (ADP-forming) alpha subunit | | 02014 | 6.2.1.5 | K01902 | 2196.7 | 0.21 | 2.12 | 2 |
| *sdhC* | Succinate dehydrogenase, cytochrome b subunit | | 01830 |  | K00241 | 820.2 | 1.26 | 2.96 | 1 |
| *sdhA* | Succinate dehydrogenase, flavoprotein subunit | | 01831 | 1.3.5.4 | K00244 | 937.2 | 1.28 | 2.99 |  |
| *sdhB* | Succinate dehydrogenase, iron-sulfur subunit | | 01832 | 1.3.5.1 | K00240 | 1447.7 | 1.12 | 2.73 | 1 |
| *fumB1* | Fumarate hydratase subunit beta | | 00293 | 4.2.1.2 | K01678 | 742.9 | -0.28 | 0.46 |  |
| *fumA1* | Fumarate hydratase subunit alpha | | 00294 | 4.2.1.2 | K01677 | 593.5 | -0.45 | 0.21 | 1 |
| *fumB2* | Fumarate hydratase subunit beta | | 02411 | 4.2.1.2 | K01678 | 592.2 | -1.45 | -1.42 |  |
| *fumA2* | Fumarate hydratase subunit alpha | | 02412 | 4.2.1.2 | K01677 | 353.1 | -1.21 | -0.92 |  |
| *mdh* | Malate dehydrogenase | | 01715 | 1.1.1.37 | K00024 | 1307.0 | -0.73 | -0.23 |  |
|  |  | **Reverse acetyl-CoA pathway** | | | | | | | |
| *codh* | Bifunctional carbon-monoxide dehydrogenase/acetyl-CoA synthase catalytic subunit | | 00607 | 1.2.7.4 | K00198 | 1049.2 | -2.45 | -1.47 | 5 |
| *cooF* | Carbon-monoxide dehydrogenase iron sulfur subunit | | 00643 |  |  | 868.2 | 0.09 | 2.06 |  |
| *acsE* | 5-methyltetrahydrofolate-homocysteine methyltransferase | | 01178 | 2.1.1.13 | K00548 | 279.7 | 0.90 | 1.32 |  |
| *mthfr* | Bifunctional homocysteine S-methyltransferase/5,10-Methylenetetrahydrofolate reductase | | 02074 | 1.5.1.54 | K00297 | 232.9 | -0.04 | 2.45 |  |
| *mthfd* | Methylenetetrahydrofolate dehydrogenase | | 02229 | 1.5.1.5 | K01491 | 442.9 | 0.39 | 2.15 |  |
| *fthd1* | Formyltetrahydrofolate deformylase | | 00419 | 3.5.1.10 | K01433 | 226.1 | / | / |  |
| *fthd2* | Formyltetrahydrofolate deformylase | | 00420 | 3.5.1.10 | K01433 | 256.0 | / | / |  |
| *fdh1* | Putative formate dehydrogenase SA2102 | | 01403 | 1.17.1.9 |  | 64.3 | -0.02 | 1.08 |  |
| *fdh2* | Formate dehydrogenase subunit alpha | | 01404 | 1.17.1.9 | K00123 | 74.8 | 0.24 | 3.17 |  |

‘/’ means data is not available, as the TPM expression values of specific genes for previous enrichment cultures are 0.

**Supplementary Table 4** Genes and proteins related to nitrate reduction in ‘*Ca.* A. nitratireducens’. The TPM expression values of *Ca.* A. nitratireducens’ in the reactor of this study were compared with Stage 1 and 2 of previous study.

| **Gene** | **Feature of gene product** | **Locus Tag** | **EC**  **number** | **KO number** | **Transcriptome** | **Log2 ratio of TPM expression values** | | **Proteome**  **(Unique peptides)** |
| --- | --- | --- | --- | --- | --- | --- | --- | --- |
|  |  |  |  |  | **(TPM)** | **This reactor/**  **Stage 1** | **This reactor/**  **Stage 2** |  |
| *napB* | Periplasmic nitrate reductase electron transfer subunit | 00811 |  |  | 814.5 | 0.57 | / | 1 |
| *napA* | Periplasmic nitrate reductase catalytic subunit | 00812 | 1.9.6.1 | K02567 | 536.6 | 0.66 | 6.01 | 7 |
| *nrfA1* | nitrite reductase (cytochrome c-552) | 00493 | 1.7.2.2 | K03385 | 8.0 | / | 1.00 |  |
| *nrfH1* | cytochrome c nitrite reductase small subunit | 00494 |  | K15876 | 11.4 | / | / |  |
| *nrfH2* | cytochrome c nitrite reductase small subunit | 01415 |  | K15876 | 48.0 | -2.59 | -3.50 |  |
| *nrfA2* | nitrite reductase (cytochrome c-552) | 01416 | 1.7.2.2 | K03385 | 92.8 | -1.70 | -2.47 |  |
|  | Putative nitrite reductase (cytochrome c-552) | 01839 |  |  | 3118.4 | 2.48 | 0.68 | 2 |
| *nrfH3* | cytochrome c nitrite reductase small subunit | 01840 |  | K15876 | 1748.9 | 2.13 | 0.06 |  |
| *norB1* | nitric oxide reductase subunit B | 00401 | 1.7.2.5 | K04561 | 28.9 | / | / |  |
| *norB2* | nitric oxide reductase subunit B | 01691 | 1.7.2.5 | K04561 | 316.4 | -1.66 | 0.00 | 5 |
| *nosD* | nitrous oxidase accessory protein | 02008 |  | K07218 | 81.6 | -0.94 | -1.58 |  |
| *nosZ* | nitrous-oxide reductase | 02010 | 1.7.2.4 | K00376 | 281.8 | -1.12 | -2.43 | 1 |
| *narB1* | Assimilatory nitrate reductase | 00583 | 1.7.5.1 | K00370 | 66.2 | / | / |  |
| *narB2* | Assimilatory nitrate reductase | 00584 | 1.7.5.1 | K00370 | 580.3 | 2.69 | 1.76 |  |
| *narK* | nitrate/nitrite transporter | 01241 |  |  | 492.3 | 2.36 | 1.87 |  |
| *nirC* | nitrite transporter | 00655 |  | K02598 | 18.8 | 0.80 | / |  |
| *nirD* | nitrite reductase (NADH) small subunit | 00656 | 1.7.1.15 | K00363 | 19.8 | 1.04 | 3.93 |  |
| *nirB* | nitrite reductase (NADH) large subunit | 00657 | 1.7.1.15 | K00362 | 20.4 | / | / |  |

‘/’ means data is not available, as the TPM expression values of specific genes for previous enrichment cultures are 0.

**Supplementary Table 5** Genes and proteins involved in anammox reactions by ‘*Ca.* Brocadia’ and ‘*Ca.* Kuenenia’.

|  | **Gene** | **Feature of gene product** | **Locus Tag** | **EC**  **number** | **KO number** | **Transcriptome** | **Proteome**  **(Unique peptides)** |
| --- | --- | --- | --- | --- | --- | --- | --- |
|  |  |  |  |  |  | **(TPM)** |  |
| ‘*Ca.* Brocadia’ | *hao1* | Hydroxylamine oxidoreductase | 01756 | 1.7.2.6 | K10535 | 232.5 |  |
|  | *hao2* | Hydroxylamine oxidoreductase | 00805 | 1.7.2.6 | K10535 | 3638.6 | 3 |
|  | *hzs* | Hydrazine synthase subunit alpha | 03058 | 1.7.2.7 | K20934 | 59418.0 | 14 |
|  | *hdh1* | Hydrazine dehydrogenase | 01804 | 1.7.2.8 | K20935 | 1461.1 | 1 |
|  | *hdh2* | Hydrazine dehydrogenase | 02887 | 1.7.2.8 | K20935 | 10958.0 | 3 |
|  | *nxrA* | Putative nitrite oxidoreductase subunit alpha | 01949 | 1.7.99.- | K00370 | 3143.5 | 4 |
|  | *nxrA* | Nitrite oxidoreductase subunit alpha | 02774 | 1.7.99.- | K00370 | 88.3 |  |
|  | *nxrB* | Nitrite oxidoreductase subunit beta | 02775 | 1.7.99.- | K00371 | 422.5 |  |
| ‘*Ca.* Kuenenia’ | *hao1* | Hydroxylamine oxidoreductase | 00242 | 1.7.2.6 | K10535 | 75.5 |  |
|  | *hao2* | Hydroxylamine oxidoreductase | 00740 | 1.7.2.6 | K10535 | 6543.7 | 2 |
|  | *hao3* | Hydroxylamine oxidoreductase | 01255 | 1.7.2.6 | K10535 | 2865.1 | 3 |
|  | *hdh* | Hydrazine dehydrogenase | 02282 | 1.7.2.8 | K20935 | 24324.6 | 4 |
|  | *nirS* | Nitrite reductase | 01508 | 1.7.2.1 | K15864 | 584.9 | 3 |
|  | *nxrB* | Nitrite oxidoreductase subunit beta | 00882 | 1.7.99.- | K00371 | 6416.6 | 5 |
|  | *nxrA* | Nitrite oxidoreductase subunit alpha | 00885 | 1.7.99.- | K00370 | 7921.3 | 8 |
